# The FAIR Case for A Coherent Interoperability to support NHS 10 Year Health Plan

**DOI:** 10.1038/s41597-026-07858-0

**Published:** 2026-07-20

**Authors:** Hanida Tomlinson, Tim Beck, Fortunato Dalrymple Castillo, Lei Clifton, Kathrin Cresswell, Rachel Dunscombe, John Farenden, Pritesh Mistry, Minal Patel, Andrew Soltan, Philippe Rocca-Serra, Susanna-Assunta Sansone

**Affiliations:** 1 Oxford e-Research Centre, Department of Engineering Science, University of Oxford, Oxford, UK; 2 Transformation Directorate, NHS England, London, UK; 3 Centre for Health Informatics, School of Medicine, University of Nottingham, Nottingham, UK; 4 British Computing Society (BCS), Swindon, UK; 5 Agile Health Informatics Ltd, London, UK; 6 Nuffield Department of Primary Care Health Sciences, University of Oxford, Oxford, UK; 7 Usher Institute, University of Edinburgh, Edinburgh, UK; 8 Institute of Global Health Innovation, Imperial College London, London, UK; 9 Health Level Seven International, Ann Arbor, USA; 10 INTEROPen, Portsmouth, UK; 11 Red Kite Health Consulting, Harrogate, UK; 12 The Kings Fund, London, UK; 13 Department of Oncology, University of Oxford, Oxford, UK; 14 Oxford University Hospitals NHS Foundation Trust, Oxford, UK

## Abstract

There is an urgent need to improve the data infrastructure supporting access to patient information within the National Health Service (NHS), England’s publicly funded healthcare system. Delivering effective, patient-centred care depends on timely access to accurate and sufficient patient information. However, achieving this remains challenging in a fragmented health and care environment, where a longitudinal patient record depends on interoperable information sharing across organisational and geographical boundaries. The health and care landscape in England is characterised by considerable organisational, technical, and governance complexity, resulting in substantial variation in digital maturity and information sharing capability across health and care settings. Within this heterogeneous environment, effective interoperability depends not only on the ability to exchange data but also on the alignment of standards, governance, and organisational capability. These interdependencies suggest that achieving coherent interoperability requires more than isolated technical solutions. Rather, they highlight the need for a unifying framework for interoperability that integrates standards, governance, and implementation, informed by internationally recognised standards and approaches. Such a framework could help shift the focus from recurring debates and isolated initiatives towards more consistent, scalable, and sustainable interoperability. Notably, current research provides limited guidance on how data governance and standards stewardship can be coordinated to achieve coherent interoperability across direct care systems. Existing approaches have largely focused on technical integration and standards implementation, with comparatively less attention has been given to the governance, semantic, and lifecycle processes required to sustain interoperability at system scale. This paper explores the role of data and standards management as foundational components of interoperability and considers the potential of the FAIR (*Findable, Accessible, Interoperable, Reusable*) Principles as a framework for advancing interoperability in direct care. Drawing on FAIR-aligned health research initiatives, we suggest that extending FAIR-informed approaches from health research to direct care within the NHS could provide a unifying framework for more coherent interoperability, while informing future standards governance, policy, procurement, and legislation in support of the NHS 10-Year Health Plan (2025–2035).

## Introduction

Effective clinical decision-making and delivery of patient-centred care requires access to timely, accurate and sufficient patient information to support better diagnosis, prevention and improved patient outcomes^1–3^. As patients often receive care from multiple care providers across primary, secondary, community, mental health, and social care settings, a longitudinal view of health records that can be shared across organisational boundaries is essential for continuity of care and informed clinical decision-making^4–6^.

Although the National Health Service (NHS) in England holds extensive health data, these remain distributed across heterogeneous clinical and administrative systems with varying levels of digital maturity^2,6^. Limited interoperability across organisational and geographical boundaries fragments care, constrains clinical decision-making, reduces operational efficiency, and can compromise patient safety^7,8^. Such fragmentation can adversely affect patient experience and trust, contributing to frustration and placing additional burdens on individuals who may be required to repeatedly recount their medical history, including distressing or traumatic experiences^8^. It also wastes clinical time and limits the delivery of patient-centred digital services, such as NHS Online^9^ and Virtual wards^10^.

The NHS 10-Year Health Plan (2025) places unprecedented emphasis on harnessing interoperable health data to support system-wide transformation, including the proposed Single Patient Record (SPR), alongside advances in artificial intelligence, genomic medicine, and wearable technologies^1,11^. Realising these ambitions requires the secure and consistent flow of health information across diverse systems, digital platforms, and organisational boundaries.

However, the ambition for health data to follow patients across care settings is longstanding and has proved difficult to realise in practice. More than two decades ago, on the 50^th^ anniversary of the NHS, the Prime Minister at the time articulated a vision for seamless access to patient information across geographical boundaries —”*If I live in Bradford and fall ill in Birmingham, I want the doctors who treat me to be able to have access to my records*”, an ambition that ultimately led to the launch of the National Programme for IT (NPfIT)^12^. Its subsequent discontinuation demonstrated the challenges of implementing a single, nationwide digital solution across England’s complex health and care system^7,13^.

Since then, national efforts have increasingly focused on connecting existing systems through interoperable interfaces rather than replacing them, reflecting a more pragmatic approach to information sharing across England’s fragmented health and care landscape^5,7,14^. Interoperability, as defined by the Healthcare Information and Management Systems Society (HIMSS)^15^ and the World Health Organisation (WHO), involves the timely, automated exchange of data across systems to improve outcomes. However, despite more than two decades of national investment in digital transformation and successive interoperability initiatives, information sharing across organisations remains inconsistent, limiting timely access to patient information and compromising care quality, operational efficiency, and patient safety^2,4,8^.

## Current Challenges

Recent policy reviews have reaffirmed persistent data interoperability challenges within the NHS. The Lord Darzi Report highlights these challenges and calls for comprehensive reforms to facilitate better data integration across healthcare service, while government investigation reports indicate that limited interoperability continues to hinder data access and integration, compromising patient safety and clinical decision-making^16,17^. These challenges were amplified during the COVID-19 pandemic, where data silos restricted timely access to critical information^18,19^.

### Fragmentation of health and social care systems

A persistent challenge across the NHS is why “seamless” data sharing remains difficult despite more than two decades of national investment and policy initiatives^17^. Observations from the literature suggest that this reflects, in part, the fragmented structure of England’s health and care system, where diverse organisations operate with differing responsibilities, procurement models, digital maturity, and governance arrangements^7,20^. The organisational tiers of care provision and their differing clinical system requirements are summarised in Table 1.

**Table 1:** Category of health and care providers in England.

Care provider	Purpose of Care
Primary care	First point of contact for most health needs e.g. General practice, Dental
Secondary care	Specialist care usually following a referral from a GP or another healthcare provider e.g. diagnostics, planned surgeries
Tertiary care	Highly specialised care for complex or rare conditions. e.g. Neurosurgery, Transplant, Plastic Surgery
Community care	Healthcare services delivered close to home or in the home e.g. District Nursing, Child Health Service, Sexual Health Services, Health visiting.
Social Care	Non-medical support for daily living and long-term needs e.g. care homes
Urgent and Emergency Care -	Immediate care for serious or life-threatening conditions e,g. Accident & Emergency, Ambulance Services (999), NHS 111
Mental Health Services	Specialist psychiatric and psychological care e.g. Mental health trusts

As of mid-2025, England’s health system comprised over 200 NHS trusts, 10 ambulance trusts, and around 6,200 GP practices, operating across a fragmented care landscape^21^. To improve coordination, 42 Integrated Care Systems (ICSs) were established in 2022 and are scheduled to consolidate into 26 clusters from April 2026^22^. ICSs are geographically defined partnerships that coordinate health and care providers within local populations to improve population health, reduce inequalities, and deliver integrated services^5,7^.

As these health and care organisations typically operate within the boundaries of their specific care functions and geographical boundaries, data exchange is limited, complicating efforts to deliver integrated care. This fragmentation is reflected in the use of heterogeneous software platforms and variable data capture practices, which continue to impede cross-organisational interoperability^6,23,24^. The literature further indicates that these challenges extend beyond technology, encompassing organisational, social, political, and economic factors^25–28^.

### Limitations in data interoperability and information exchange

Although interoperability is often positioned as a key enabler of data exchange to improve outcomes, its effectiveness will depend on the ‘*operability*’ of data sources^29^ and the ‘*co-operability*’ of organisations^3^ within the interoperability ecosystem. There is complex interplay between policy frameworks, inter-organisational arrangements, and interoperability constraints in the implementation of integrated care infrastructure across the NHS^7^. Also, high quality data appear to be contextual and vary according to the purpose of data sharing and therefore may depend on effective, operable source systems, and sustained ‘*co-operability’* to support consistent data and standards practices.

**Fig. 1:**
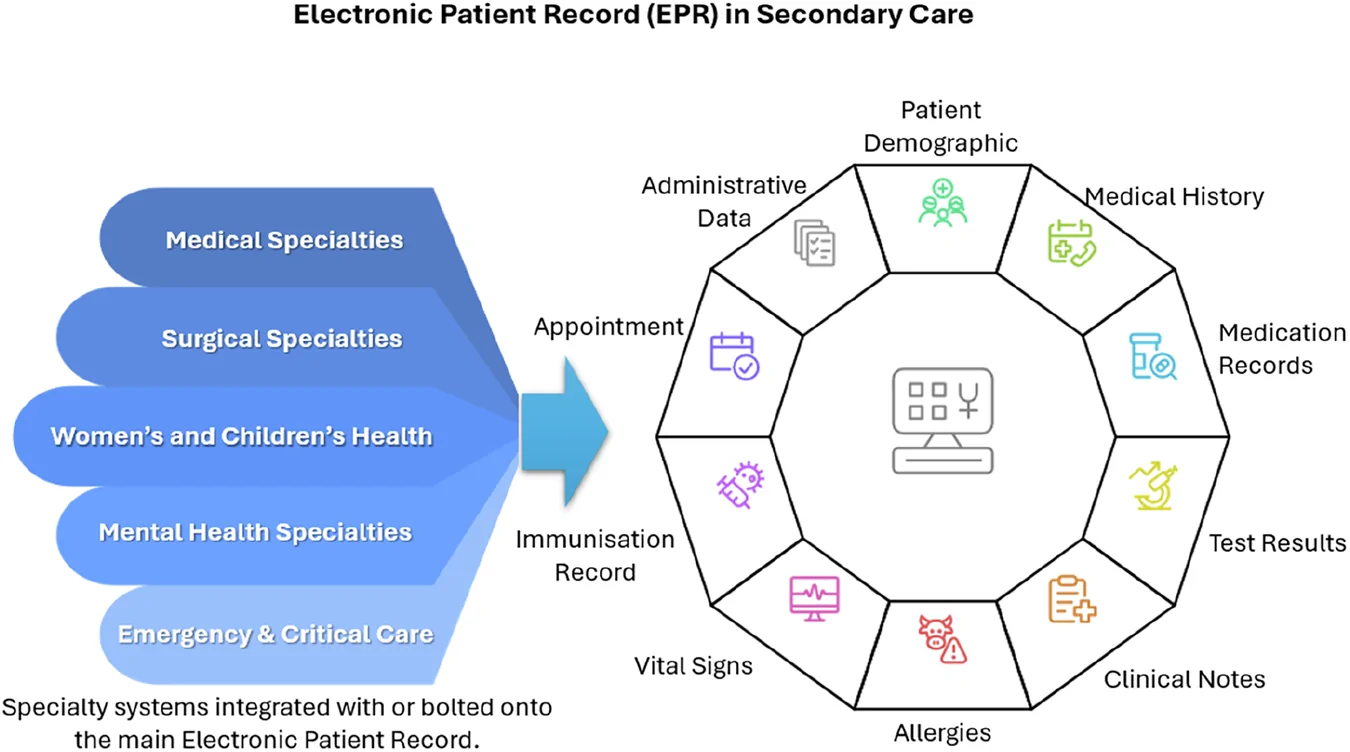
Illustration of intra-organisational interoperability, showing the need for information exchange between clinical systems across specialties within a single provider.

Li *et al*.^2^ identify two key types of interoperability: intra-organisational and inter-organisational. Intra-organisational interoperability refers to information sharing within a single care provider; this may involve any of the care providers listed in Table 1, supporting coordination and integration of multiple internal services, as illustrated in Fig. 1.

**Fig. 2:**
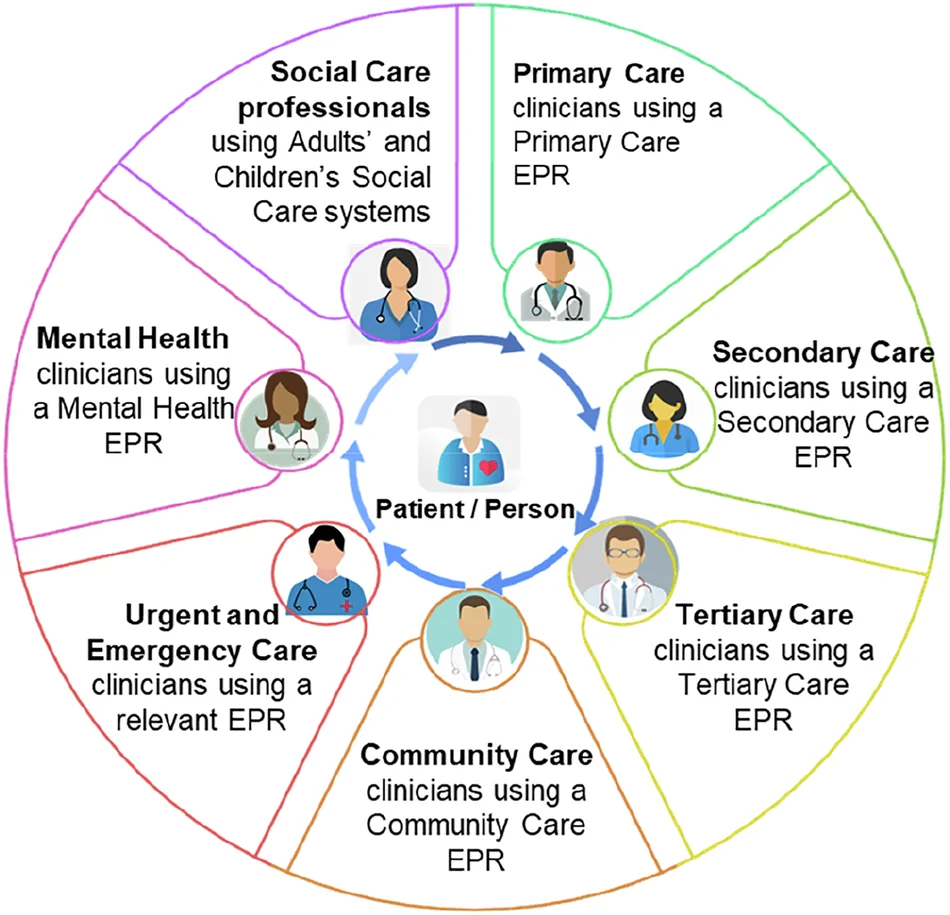
Illustration of Inter-organisational interoperability: showing the need for information exchange between clinical systems across care providers to achieve a unified, longitudinal view of patient records wherever patients receive care.

Across a patient’s care pathway, individuals may engage with some, or all the care providers listed in Table 1. Inter-organisational interoperability involves the exchange of data between different care providers as illustrated in the patient journey shown in Fig. 2.

Inter-organisational interoperability refers to information exchange across care providers to support a unified, longitudinal view of patient records, regardless of where care is delivered. Achieving this depends on integrating data and fostering ‘*co-operability’* across clinical systems, organisations, and geographical boundaries. This capability is widely recognised as a key enabler of patient-centred (or person-centred) care^5^, particularly in specialist and urgent services that operate across multiple ICSs, such as ambulance services, where timely access to relevant patient information is critical to safe and effective care.

### Inconsistent access to patient information at the point of care

Despite continued investment, inconsistent information exchange continues to limit access to complete patient information and informed clinical decision-making, causing suboptimal care experiences for patients^8,30,31^. Multiple parallel initiatives and heterogeneous clinical systems often require clinicians to access information through multiple interfaces, making it difficult to establish the most current and complete patient record. Information sharing may be further constrained by data governance and controllership arrangements^7,20^, particularly where legal accountability for data breaches or negligence in shared data environments is uncertain. Product design choices and the cost of interoperable capabilities may also contribute to information blocking, limiting the scalability of interoperability initiatives.

### Patient experience and burden of repeated sharing of health information

Patients report a sense of disconnection when navigating multiple health and care providers, often having to repeatedly share the same personal health information during care transitions^8,30^. This may compromise data accuracy and place the burden on patients to bridge information gaps through informal workarounds^8^. Coroners’ reports have also identified cases where limited access to patient information contributed to adverse outcomes^2^. Due to this, public feedback on the 10-Year Health Plan has highlighted optimism about the potential of a single patient record to improve continuity of care^32^.

### Work to date and standing issues

Successive national strategies, including the *Five Year Forward View* (2014), *NHS Long Term Plan* (2019), *Data Saves Lives* (2021), the *Goldacre Review* (2022), and the *10 Year Health Plan* (2025) have consistently highlighted the importance of high-quality, interoperable health data for integrated care and timely access to patient information. A National Audit Office (NAO) report similarly describes interoperability across health data and IT systems as a long-standing objective, while highlighting tensions between improving interoperability and increasing supplier diversity due to the complexity of system integration^17^. Although a draft NHS Standards and Interoperability Strategy was developed and consulted on in 2022^33^, it was not published. England, and the wider United Kingdom, therefore, appear to lack a comprehensive national interoperability strategy comparable to Australia’s *National Healthcare Interoperability Plan*^34^ or the *European Interoperability Framework*^35^, limiting a shared strategic vision and guiding principles for coordinating interoperability efforts across the health and care system.

Over the past two decades, several national initiatives have been introduced to improve information sharing across the NHS, including the Summary Care Record (SCR), GP Connect, the Shared Care Record (ShCR), Connecting Care Records (ConCR), National Care Records Service (NCRS), and the Federated Data Platform (FDP). While these programmes have advanced interoperability within specific care settings, differences in scope, implementation, technical maturity, and priorities across initiatives continue to limit coherent interoperability and a longitudinal view of patient records. Multiple data sharing initiatives do not necessarily lead to better data sharing^18^. Moreover, implementation has often prioritised technical solutions, while organisational factors such as leadership, collaborative working, access to funding for digital transformation, and the development of skills and capabilities, are often less explicitly addressed^7,25^.

Data coherence depends on effective coordination across care providers within a patient’s care ecosystem and is reflected in the consistent use of agreed datasets to support a longitudinal view of patient records^3^. As for ‘*interoperability coherence*’ we mean the degree of coordination, alignment and synergies between the initiatives. Within the NHS, interoperability initiatives have often progressed in parallel, with limited evidence of systematic alignment across programmes. For example, the Connecting Care Record (ConCR), introduced in 2023 to support cross-boundary data sharing using the International Patient Summary standard, and the Federated Data Platform (FDP)^36^, rolled out nationally from 2024 to connect data across NHS organisations, pursue complementary objectives but have largely evolved as separate initiatives within the wider data architecture.

In addition, the notion of “*seamless*” interoperability frequently used in NHS policy and implementation programmes^1,17^, remains open to interpretation. At one end of the spectrum, it may describe basic information exchange, such as sharing PDF discharge summaries; at the other, it may imply real-time access to structured, longitudinal patient records across systems, organisations, and geographical boundaries. This ambiguity can create unrealistic expectations and obscure the technical, organisational, and governance challenges required to achieve meaningful interoperability.

How data are generated, shared, and used reflects the processes, organisational models, and regulatory and policy frameworks that govern the system^3^. Consequently, interoperability challenges arise from governance and structural constraints as much as from technical limitations. Interviews with NHS Chief Clinical Information Officers highlight the need for common data standards to address persistent interoperability challenges^2^. Current approaches, including the NHS Standard Contract^37^, and procurement-led mechanisms^38^ seek to promote standards adoption through commissioning and purchasing requirements. However, implementation remains uneven across programmes, reflecting differences in system complexity, procurement priorities, and investment. More stringent requirements are typically applied to large-scale systems such as electronic patient records (EPRs) than to smaller specialty systems, while long contract durations may further constrain system-wide improvement.

## Discussion

### The need of unified standards framework to improve interoperability

To date, many interoperability initiatives have been driven by specific use cases and operational needs. While valuable, these efforts have often evolved independently, contributing to a fragmented and inconsistent information standards landscape. Consistent with this observation, Li *et al*.^2^ and de Mello *et al*.^39^ highlight continued variation in terminology, classification, and data exchange standards. The resulting mix of legacy, emerging, and nationally mandated standards, together with uneven implementation, continues to impede system-wide interoperability.

Against this backdrop, the NHS requires more than additional standards or technologies; it requires a unifying standards framework that aligns the technical, semantic, organisational, and governance dimensions of interoperability. Such alignment is essential to achieving coherent interoperability, which refers to the coordinated alignment and lifecycle stewardship of standards, metadata, semantic models, and governance processes across organisational boundaries. Such alignment could support more consistent exchange, interpretation, and reuse of health data while strengthening provenance and reducing duplication across the care pathway.

**Fig. 3:**
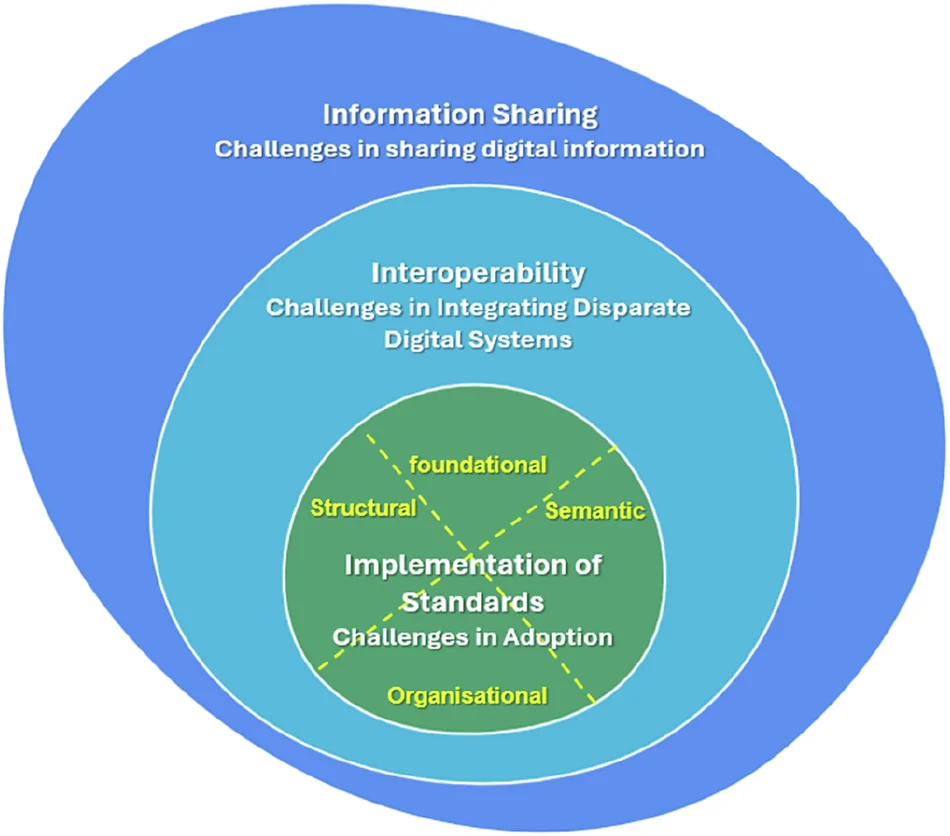
Illustrates the layered structure of underlying causal factors affecting information sharing for integrated care, with data standards representing a core systemic challenge to achieving interoperability.

This perspective points to the need for a nationally led approach to interoperability and data accessibility to support the ambitions of the10-Year Health Plan^1^ and the Life Sciences Sector Plan^40^. Effective information exchange depends on standardised formats, consistent syntax, and aligned organisational practices^39^, while also requiring flexibility to accommodate diverse health IT systems^41^. HIMSS defines four levels of interoperability — foundational (basic data exchange), structural (syntactic consistency), semantic (shared understanding), and organisational (governance and policy)^15,42^. Collectively, these four interoperability levels highlight that interoperability is not a single capability but a set of interconnected layers, each presenting distinct challenges that require coordinated standards implementation^43^. Figure 3 illustrates how standards, interoperability, and information-sharing challenges are interrelated.

Observations from international health systems and other sectors suggest that achieving system-wide interoperability may require stronger national mechanisms to support standards adoption^2,41,44^. In response, the Department of Health and Social Care and NHS England appear to be exploring a more legislative approach, drawing lessons from sectors like banking and retail, where mandatory interoperability standards have successfully enabled both innovation and consistency. International examples, including United States through the *21*^*st*^ *Century Cures Act*^44^, Finland, Taiwan, and Denmark^41^, suggest that legislative approaches may support more consistent implementation of health data standards. In England, this direction is reflected in two legislative measures that are expected to support this shift — Section 250 of the *Health and Care Act 2022*, which provides powers to mandate compliance with information standards by health and social care providers^45^, and the *Data (Use and Access) Act 2025*, which extend these provisions to IT system suppliers^46^.

However, mandating standards at scale, particularly within legacy infrastructures, can create tension between national requirement and local capabilities. Although the HIMSS interoperability model defines the capabilities required across foundational, structural, semantic, and organisational levels, it provides limited guidance on how these should be aligned, governed, measured, and sustained over time. Achieving coherent interoperability is therefore likely to require complementary governance principles that align implementation across all four levels.

### Why does this matter

Drawing on the health and care landscape in England described above, it is inevitable that data for direct care will continue to operate within a heterogeneous information environment. Fragmented connectivity across health systems transfers coordination burdens to patients and clinicians, strains service delivery, and undermines continuity, safety, and the overall experience of care^8,30^. These issues are likely to be further amplified by the growing adoption of artificial intelligence (AI) enabled solutions in NHS such as Ambient Voice Technology (AVT)^47^ and predictive analytics/omics, which place additional demands on robust and well-governed data foundations. A growing body of evidence indicates that unified information standards are essential for scalable and effective data exchange within and across health systems^2,8,41^. Beyond facilitating exchange, good data management is a key prerequisite to discovery and innovation^48^. Such management depends on agreed standards that are essential for making data usable by both humans and machines^18,19,49^.

NHS England and the Department of Health and Social Care define information standards as specifications governing the design, quality, interoperability, accessibility, portability, and security of health information systems^45,46^. A National Audit Office report indicated that earlier approaches to standards implementation were perceived by stakeholders as adding complexity rather than improving interoperability, resulting in the use of multiple standards or divergent versions of the same specification. The report also observed that reported compliance with clinical terminology standards among trusts remained low^17^.

Despite stakeholder calls for co-developed standards and alignment with clinical practice^2,8,19^, evidence on how to effectively design, implement and govern system-wide frameworks for interoperability, and to rationalise, simplify, and standardise the standards landscape, remains limited. It is also worth noting that while developing standards is comparatively straightforward, their implementation, adoption, and long-term stewardship present far greater challenges.

High quality, standardised, and accessible data are essential to realising the ambitions of the 10-Year Health Plan and the delivery of integrated, patient-centred care. Their importance will only increase as AI and other data-driven technologies become more widely adopted, placing greater demands on reliable, machine-readable, and well-governed data. Yet, despite substantial investment in standards, digital infrastructure, and interoperability initiatives, fragmentation persists. This suggest that the challenge is not the absence of standards, but the lack of coordinated governance and stewardship across the standards ecosystem Addressing this will require a unified framework that aligns governance, semantics, and lifecycle stewardship to support coherent interoperability as an integrated system capability.

### The case for FAIR data management approaches for direct care

Fragmented and poorly interoperable data infrastructures also impede information flows and limit data reuse in research settings^48,50,51^. The Sudlow Review similarly emphasises the need for overcoming current interoperability barriers to realise the vast potential of unified health data to benefit society^52^.

In the research and academic domain, particularly within the life sciences, significant efforts have already been directed towards addressing data challenges that closely mirror those faced by health systems such as the NHS. The FAIR guiding principles, introduced in 2016, promote good data stewardship and the infrastructure needed for reliable data reuse and integration by both humans and machines^48,50,53^. They were developed in response to persistent challenges in research data management, including fragmented data ecosystems, limited interoperability across communities and repositories, and inconsistencies between datasets^48,50^. These challenges are further characterised by limited data discovery and reuse^43^, insufficient machine actionability^48,50,51^ and the absence of clearly shared criteria for good data management^43,53^. Notably, similar limitations are observed in direct care, where health data are distributed across heterogeneous systems, encoded inconsistently, and often insufficiently structured for automated processing.

FAIR related studies and output to date have been most extensively developed within research and large-scale data analytics across multiple sectors, particularly in the life sciences and biomedical research^48,51,54^ as well as health informatics^18,19,53^, where data must be shared, trusted, and reused across organisational and disciplinary boundaries. FAIR principles have seen increasing adoption within European research initiatives and in the UK, by organisations such as Health Data Research UK (HDRUK).

A growing body of FAIR-aligned methods and research outputs including implementation guidance such as the FAIR Cookbook^54^, domain-specific solution and workflows such as FAIR4Health^53,55^. Extending FAIR principles to healthcare data governance and infrastructure could improve machine readability, semantic interoperability, and data quality across both health research and direct care. However, their application in routine direct care in England remains limited, and current record management practices may not yet fully reflect the distinct requirements of digital health records.

To date, FAIR implementations have largely focused on research reproducibility, secondary data use, and machine-based discovery. Examining how these principles could be adapted and operationalised for direct care within the NHS would provide insight into their applicability beyond research. Aligning FAIR approaches across health research and direct care could promote more consistent data stewardship, interoperability, and trust across a shared health data ecosystem, recognising that both domains rely on data derived from the same patients and populations.

### Implementation considerations

Substantial groundwork for interoperability and standards management has already been established within the NHS. Therefore, addressing these challenges do not necessarily require starting from scratch or pursuing radical new solutions, but rather the systematic reorganisation, alignment, and rationalisation of existing standards and initiatives. Existing resources, including the Interoperability Toolkit^56^, Open API Policy^57^, NHS Standards Directory^58^, and the NHS API Catalogue^59^ may be regarded as incremental steps towards a more coordinated standards ecosystem. Building on these foundations will require a clearer, system-wide approach to standards governance, with shared principles and practical guidance to support consistent implementation across the NHS.

Table 2 illustrates the conceptual relationship between the HIMSS interoperability model and the FAIR principles. In this conceptualisation, the HIMSS model defines four interoperability levels — foundational, structural, semantic, and organisational, while FAIR provides cross-cutting principles that could support their coordinated implementation. Together, they provide a conceptual basis for coherent interoperability through the alignment of standards, semantic models, governance, and lifecycle stewardship across organisational boundaries.

**Table 2:** Cross-mapping matrix between FAIR principles (columns) and HIMSS interoperability levels (rows).

FAIR and HIMSS Interoperability Matrix				
	FAIR Principles			
**HIMSS Level**	Findable (F) *Data and metadata should be easy to locate for both humans and machines*	Accessible (A) *Once found, data should be retrievable using standardized protocols*.	Interoperable (I) *Data should be compatible with other datasets and tools*.	Reuseable (R) *Data should be well-described and documented to allow for replication and further use*
**Foundational Interoperability** *Basic data exchange*	F1. (meta)data are assigned a globally unique and persistent identifier F4. (meta)data are registered or indexed in a searchable resource	A1. (meta)data are retrievable by their identifier using a standardized communications protocol A1.1 the protocol is open, free, and universally implementable A1.2 the protocol allows for an authentication and authorization procedure, where necessary	Limited contribution; establishes connectivity rather than meaning	Limited contribution: data exchange possible but reuse not guaranteed
**Structural Interoperability** *Consistent data formats and structure*.	F2. data are described with rich metadata (defined by R1) F3. metadata clearly and explicitly include the identifier of the data it describes	A1. (meta)data are retrievable by their identifier using a standardized communications protocol A2. metadata are accessible, even when the data are no longer available	I1. (meta)data use a formal, accessible, shared, and broadly applicable language for knowledge representation.	R1.3. (meta)data meet domain-relevant community standards
**Semantic Interoperability** *Shared meaning of the data*.	F2. data are described with rich metadata (defined by R1)	Metadata provides contextual understanding for retrieval and use	I1. (meta)data use a formal, accessible, shared, and broadly applicable language for knowledge representation. I2. (meta)data use vocabularies that follow FAIR principles I3. (meta)data include qualified references to other (meta)data	R1. meta(data) are richly described with a plurality of accurate and relevant attributes R1.3. (meta)data meet domain-relevant community standards
**Organisational Interoperability** *Collaboration, policy alignment, and real-world use of data*.	Governance and stewardship of registries and catalogues	A1.2 the protocol allows for an authentication and authorization procedure, where necessary A2. metadata are accessible, even when the data are no longer available	Governance alignment supporting semantic consistency across organisations	R1.1. (meta)data are released with a clear and accessible data usage licence R1.2. (meta)data are associated with detailed provenance R1.3. (meta)data meet domain-relevant community standards

Cells indicate FAIR sub-principles that contribute to each layer. FAIR principles are not layer-specific; rather, different sub-principles may apply across multiple layers, reflecting FAIR’s role as a cross-cutting governance framework.

Although coherent interoperability remains an emerging concept, its implementation could be assessed through three dimensions: (1) Standards version synchronisation rate, reflecting technical alignment; (2) Metadata machine-readability scores, ensuring semantic consistency; (3) Cross-organisational governance coordination, supporting sustainable data collaboration. Notably, FAIR should not be viewed as another interoperability standard. Rather, it complements existing standards ecosystems by providing principles for the governance, stewardship, and lifecycle management of data and metadata, whereas standards such as HL7 FHIR^60^, SNOMED CT^61^, DICOM^62^, and ISO^63^ define how information is structured, exchanged, and interpreted.

FAIR-related research has developed a range of frameworks, tools, and evaluation approaches that may have relevance beyond research settings. Given shared data management challenges across research and direct care, FAIR-informed outputs and workflows including, FAIRification framework^49^, the FAIR Cookbook^54^, community-driven approaches to assessing FAIRness^55^, and machine-actionable metadata models^64^, offer useful starting points for adaptation within direct care in the NHS. Collectively, these approaches could support a more structured and sustainable approach to data management and interoperability across the wider health and care ecosystem.

The literature identifies recurring interoperability challenges across both health research and direct care. Organised around the four interoperability levels of the HIMSS model, the following sections examine how FAIR principles could help address these challenges at each level.

#### Advancing foundational interoperability

The foundational interoperability level describes the capability of systems to securely exchange data, enabling automated information transfer without requiring semantic interpretation.

Within the NHS, foundational interoperability remains constrained by fragmented data distributed across heterogeneous systems. Variation in standards, versions, vocabularies, and the growing use of digital health technologies complicate the consolidation and exchange of patient information. As this heterogeneity is likely to persist, effective data sharing will depend on a common data architecture capable of supporting diverse systems^41^.

Comparable challenges exist in research and large-scale analytics, where institutional data silos limit interoperability and data-intensive science^50,51,65^. FAIR has emerged as a widely adopted framework for supporting data exchange across distributed research infrastructures through persistent identifiers, searchable metadata, and standardised access mechanisms^48,50^.

As shown in Table 2, FAIR could strengthen this level through persistent identifiers (F1), searchable registries (F4), and standardised access mechanisms (A1). Together, these principles could improve the discoverability, accessibility, and reliable exchange of data and services across distributed systems. Although FAIR has not yet been widely adopted in direct care, these principles could help guide more consistent approaches to connectivity across heterogeneous health information systems.

#### Advancing structural interoperability

The structural interoperability level describes the syntactic consistency achieved through standardised data formats, structures, and exchange protocols, enabling receiving systems to interpret exchanged data accurately.

Within the NHS, structural interoperability remains constrained by inconsistent implementation of standards. Although common standards such as HL7 FHIR, SNOMED CT, DICOM, and ISO are widely used, their concurrent adoption does not necessarily result in consistent implementation. For example, variation in HL7 FHIR profiles contributes to differences in interpretation across systems. Evidence suggests that standards achieve greater uptake when they are streamlined, aligned with clinical workflows, and supported by clear implementation guidance^41^. Without mechanisms to coordinate implementation across standards, divergence in practice is likely to persist, limiting data harmonisation and reuse.

Comparable challenges exist in research and data-intensive environments, where fragmented standards development, inconsistent metadata, and variable implementation constrain interoperability and machine actionability^19,54^. In both settings, inconsistent implementation across standards ecosystems contributes to persistent structural heterogeneity.

Table 2 shows that structural interoperability aligns strongly with FAIR principles relating to metadata and machine-readable structures. F2, F3, A1, and I1 may help standardised representation and exchange of data, support standardised representation and exchange of data, enabling receiving systems to parse and process exchanged data consistently while reinforcing implementation of FHIR resources, profiles, and implementation guides. Through consistent metadata, implementation profiles, and machine-readable schemas, FAIR could support more consistent implementation across standards ecosystems^48,50,53^.

Emerging approaches, such as domain-specific “*FAIR profiles*”^54^ and automated FAIR metadata capture within routine clinical systems^50,54^, provide practical opportunities to strengthen structural interoperability without increasing clinical burden. Rather than introducing additional standards, FAIR could support more consistent implementation of existing standards across the NHS.

#### Advancing semantic interoperability

The semantic interoperability level describes the capability of systems to interpret, and use exchanged data consistently through shared vocabularies, data models, and value sets. By establishing common meaning across systems, it provides the foundation for reliable data reuse.

Within the NHS, semantic interoperability remains constrained by inconsistent data capture across health and social care^2,30^, compromising interoperability, data quality, and information sharing^15^. Inconsistent coding, incomplete contextual metadata, and variable data entry across NHS systems further compromise reliability and limit the value of data-driven decision-making. Consequently, outcomes may be measured inconsistently across services, highlighting the need for approaches that harmonise heterogeneous datasets and strengthen data integrity, traceability, and reproducibility across the NHS.

Table 2 highlights that the strongest synergy between FAIR and the HIMSS interoperability model occurs at the semantic level. FAIR principles I1, I2, and I3 directly reinforce semantic interoperability by promoting shared representation languages, controlled vocabularies, and explicit semantic relationships. Principles R1 and R1.3 further strengthen meaning through rich contextual metadata and adherence to domain standards such as SNOMED CT. Collectively, these principles could support the consistent interpretation of data across systems and organisations, which is fundamental to safe and integrated care.

Beyond interoperability, the FAIR Reusable principle promotes meaningful data reuse through contextual metadata, provenance, and documentation^48–50^. Resources including the FAIR Cookbook and FAIR maturity indicators provide practical approaches that could be adapted for direct care^54,55^. Together, these approaches could support semantic consistency, reliable data linkage, and reuse across heterogeneous datasets^65^.

Taken together, the evidence suggests that FAIR principles may be particularly valuable at the semantic level, where they could reduce ambiguity, support consistent interpretation across organisations, and enable consistent integration of heterogeneous datasets. Such semantic clarity is fundamental to safe, integrated care^65^. However, the translation of ontological models, including common data elements and FAIR Digital Objects remains insufficiently evaluated^19^. Further empirical investigation is therefore required to determine how these can be operationalised within clinical workflows while preserving usability, safety, and system performance.

#### Advancing Organisational Interoperability

The organisational interoperability level refers to the governance, policy, and regulatory mechanisms required to support secure and coordinated data use across organisations. In England, progress remains constrained by the absence of a coherent national standards roadmap and limited alignment with international frameworks, contributing to fragmented implementation and inconsistent governance across the health and care system^2,41^. Governance approaches have often focused on data publication rather than lifecycle stewardship, highlighting the need for governance models that support provenance, reuse, and long-term stewardship throughout the data lifecycle^49^.

FAIR principles offer a complementary approach to addressing these challenges. As demonstrated in Table 2, organisational interoperability aligns most closely with the FAIR Reusable principles, particularly through R1.1 (licensing), R1.2 (provenance), and R1.3 (community standards), which strengthen accountability, transparency, and stewardship across organisational boundaries. Resources such as FAIRsharing^66,67^, the FAIR Cookbook^54^, and the FAIR Flexible Framework^49^ offer practical approaches for embedding FAIR by design and supporting lifecycle data stewardship. More broadly, FAIR may strengthen organisational interoperability through consistent governance, provenance, and lifecycle stewardship, supporting accountable collaboration and the sustainable reuse of health data across organisational boundaries^48^.

Nevertheless, achieving FAIR data requires more than principles alone; it depends on shared infrastructure, robust governance, and coordinated community action^66^. Limited incentives and practical guidance could hinder adoption. The maturity, uptake, and implementation of standards are often difficult to assess, limiting coordinated decision-making among standards developers, repository managers, publishers, and policymakers^66^. FAIR addresses these challenges through community-led governance of metrics, maturity indicators, and assessment frameworks, promoting greater transparency and consistency^55^.

Embedding FAIR within England’s health system, in partnership with clinicians and data managers, could help align technical standards with operational realities^19,41^. Existing governance models, including the Research Data Alliance, European Open Science Cloud, World Wide Web Consortium, and Internet Engineering Task Force^55^ offer examples of transparent, community-led coordination that could inform NHS implementation. Collectively, these approaches suggest that FAIR could strengthen organisational interoperability through more accountable, lifecycle-oriented data stewardship, although further evaluation in routine health and care settings is required.

## Conclusion

Achieving coherent interoperability across England’s health and care system remains a significant challenge despite decades of policy development, standards implementation, and digital investment. Although support for common data standards continues to grow^2,41^, fragmented implementation and the absence of a unified standards framework continue to limit coordinated, system-wide interoperability. Addressing these challenges will require more than technical standards or legislative measures alone; it will depend on coordinated governance, organisational alignment, and sustained stewardship to enable interoperability as an integrated system capability^8,30^.

FAIR offers a potential cross-cutting framework for strengthening interoperability by aligning the foundational, structural, semantic, and organisational dimensions of health data management. Rather than introducing new standards, FAIR complements existing standards ecosystems through principles that support discoverability, standardised representation, semantic consistency, provenance, and lifecycle stewardship. If successfully operationalised, FAIR-informed approaches could support more coherent interoperability across direct care and health research, while contributing to the ambitions of the NHS 10-Year Health Plan and the Life Sciences Sector Plan.

Although FAIR offers a promising framework for advancing interoperability, it is unlikely to deliver meaningful change in isolation. Its successful implementation will depend on addressing the technological, organisational, cultural, and workforce challenges that shape adoption in practice, supported by evaluation in real-world settings. Likewise, interoperability across the NHS is unlikely to be achieved through procurement, contractual, or legislative mechanisms alone. Sustainable progress will require a nationally coordinated approach to standards selection, implementation, governance, and lifecycle stewardship, supported by enabling infrastructure, practical implementation guidance, and sustained collaboration between the NHS, industry, academia, and standards bodies. Future research should therefore move beyond conceptual alignment to evaluate how FAIR-informed approaches could be operationalised and whether they deliver measurable improvements in interoperability, data quality, and integrated care in routine health and care settings.

## Data Availability

No new data were generated or analysed in support of this article.
